# Ambiguities in cutaneous leishmaniasis classification and the need for consensus: Experience from Ethiopia

**DOI:** 10.1371/journal.pntd.0013458

**Published:** 2025-08-22

**Authors:** Saskia van Henten, Ermias Diro, Annisa Befekadu Tesfaye, Feleke Tilahun Zewdu, Johan van Griensven, Wendemagegn Enbiale

**Affiliations:** 1 Department of Clinical Sciences, Institute of Tropical Medicine, Antwerp, Belgium; 2 Department of General Internal Medicine, University of Washington, Seattle, Washington, United States of America; 3 Department of Internal Medicine, University of Gondar, Gondar, Ethiopia; 4 Department of Dermatovenereology, University of Gondar, Gondar, Ethiopia; 5 Boru Meda Hospital, Dessie, Ethiopia; 6 Department of Epidemiology and Biostatistics, University of Gondar, Gondar, Ethiopia; 7 Dermatovenerology Department, College of Medicine and Health Sciences, Bahir Dar University, Bahir Dar, Ethiopia; 8 Collaborative Research and Training Center for Neglected Tropical Diseases, Arba Minch University, Arba Minch, Ethiopia; Wadsworth Center, UNITED STATES OF AMERICA

## Abstract

**Background:**

Cutaneous leishmaniasis (CL) is clinically classified into localized (LCL), mucocutaneous (MCL) and diffuse (DCL) types. While conducting a treatment study on CL at two sites in Ethiopia (Boru Meda and Gondar), differences in opinion in the classification of CL became apparent. The lack of uniformly understandable classifications has made comparison and generalizability of research findings challenging. We wanted to investigate the extent of agreement in CL classification between CL experts and describe on which points there was disagreement.

**Methodology/principal findings:**

Thirteen CL experts in Ethiopia were provided with 26 high-quality photographs of CL lesions from patients enrolled in a clinical study on miltefosine treatment. Patients were selected for this sub study due to potential classification difficulties. The most common (majority vote) classification category was noted, and the proportion of experts which choose this (% agreement). Majority vote classification was used to reclassify patients compared to the original study classification. Among included patients, sixteen were originally classified as DCL, eight as MCL, one as LCL, and one as *Leishmania* recidivans. There was no unanimous consensus for any of the selected cases. The maximum agreement was 80%, which was seen for 38% (10/26) cases. Disagreements existed on whether patients had mucosal involvement, how to classify patients with singular large diffusely swollen lesions and when to classify patients with multiple lesions as DCL. According to the majority vote, 62% of selected patients would be reclassified to a different CL classification compared to the original study from which photographs were selected.

**Conclusions/significance:**

There is no clear understanding and harmony in the classification of CL. Reproducible classification guidelines and training of healthcare providers on CL are needed to ensure consistency in further classification to allow generalizability and comparison of clinical trial findings. Simpler classifications with direct links to treatment decision-making would be valuable.

## Author summary

Cutaneous leishmaniasis (CL) appears in different forms and is commonly classified into three major forms called localized CL, muco-cutaneous leishmaniasis (MCL)and diffuse CL (DCL). The decision how to treat and the expected treatment outcomes are also thought to vary depending on the type of lesion. We found out that health care providers have conflicting approaches in categorizing CL lesions into these different subgroups. We showed this difference systematically by asking experts to classify pictures taken from Ethiopian CL patients selected purposefully because of potential classification issues. Experts could not agree on classification of a single patient, with maximum agreement of 80% (10/13) for ten of the 26 cases. As consistent classification helps for comparison and experience-sharing between different CL endemic regions, and therefore healthcare providers need to be trained to harmonize the care practices. Rather than producing more complicated classification systems, such systems are ideally simple, easily applicable by clinicians and linked to clinical decision-making.

## Introduction

Cutaneous leishmaniasis (CL) is a neglected tropical skin disease, resulting in disfiguring lesions that cause significant stigma and suffering. Caused by *Leishmania* parasites, it is transmitted through the bite of female phlebotomine sandflies. CL is common in many regions around the world, with an estimated 700,000 – 1,200,000 new cases occurring yearly [1]. Different *Leishmania* species are found as causative species for CL in different regions, broadly classified as New World (NW) CL in South and Central America, and Old World (OW) CL in Europe, Asia, and Africa. Depending on the species and the host response, CL varies in terms of clinical presentation, localization of lesions, disease evolution and response to treatment. Clinically, CL is broadly classified into localized CL (LCL), mucocutaneous CL (MCL), and diffuse CL. However, there are several varieties that do not typically fall under this classification described both for OW [2,3] as well as NW CL [4,5]. These variations include *Leishmania* recidivans, sporotrichoid-like, erysipeloid-form, lupoid or eczematous type and disseminated cutaneous leishmaniasis. Some patients also present with multiple lesions, combined forms and involvement of atypical body sites making the categorization into the typical known classification challenging.

*L. aethiopica* is unique since it is almost exclusively seen in the highlands of Ethiopia. In addition, it causes a wide spectrum of clinical CL presentations, ranging from tiny papules and/or ulcers to disseminated nodular lesions. Most commonly, lesions present with erythema, diffuse swelling, plaques, and crusts, while only a minority of lesions show ulceration [6,7]. CL in Ethiopia is also similarly classified into three forms. Typical presentations of LCL (A), MCL (B) and DCL (C) are shown in S1 Fig.

Classification organizes patients into groups, assuming those within the same group are clinically more similar to each other than those in the other groups. Clearly defined and agreed classification approaches would enable comparison of research findings of similar lesions in different regions. Importantly, it also has consequences for treatment, as both MCL and DCL are recommended to be treated with systemic treatment, and many clinical trials exclude such patients from inclusion. Non-harmonized classification criteria could lead to bias in reported treatment outcomes. For instance, when clinicians classify more patients as MCL or DCL instead of LCL, it could result in inflated cure rates for the remaining (probably less-severe) LCL patients.

There is an increasing call for clinical trials on CL [8,9] in line with global efforts to improve CL treatment. As it stands, lack of standardized methodologies including classification has complicated comparison and generalizability of clinical trial findings from various parts of the world.

We conducted a study on the effectiveness of miltefosine treatment for CL at two sites in Ethiopia [7] where cure rates were found to be remarkably good for MCL (79%) and DCL (91%) which raised some questions about how classification was done. A careful review of patient photographs and discussion with other CL experts showed variations in the classification for more than a quarter (26/94, 28%) of the patients included in the study. However, these ambiguities in classification have never formally been studied. Data from this paper was used as a starting point to investigate which classifications are given for these difficult patients and to which extent there is agreement in classification. We did this by providing high-resolution patient photographs of these difficult cases to a panel of Ethiopian CL experts to independently classify and provide reasoning for the classification. This information can serve as a first step towards a consensus-backed classification of CL in the Ethiopian setting.

## Methods

### Ethics statement

The original study in which patient data was collected was approved by Institutional review board of University of Gondar and the National Ethiopian ethical review board, as well as the Institutional review board of Institute of Tropical Medicine, Antwerp, Belgium, and the University Hospital of Antwerp. Written informed consent was obtained from all participants (or from the parent/guardian for children), with additional written assent collected from children aged 12–18. Patients gave specific consent for their lesion to be photographed and for their data to be shared as long as they could not be personally identified. Photographs were de-identified by cropping out lesions whenever possible, and by blurring identifying features. Photographs were shared securely through a protected server where clinicians could open but not modify the pictures. The current sub-study in which the de-identified photos were used and supplied to CL experts received exemption from further ethical review.

### Data

Patient photographs were collected as part of a clinical study investigating the effectiveness of miltefosine in Gondar and Boru Meda, Ethiopia (NCT04004754) [7]. The initial classifications were made by treating clinicians (ABT for Gondar and FTZ for Boru Meda), with each clinician also asked to classify patients from the other study site based on photographs. This showed inconsistencies, leading to informal discussions with other physicians (WE, ED, JvG), which further highlighted classification difficulties.

Photographs from patients included in the study were purposefully selected by SvH following these discussions, focusing on the cases where classification issues were evident. One patient with a straightforward LCL classification (case QC) was included as quality control.

Information on lesion size, duration, and number of lesions were provided, as well as certain characteristics not clearly visible in photographs, for instance if the lesions were limited to a specific body part.

Clinicians involved in CL care in Ethiopia were identified by a senior Ethiopian dermatologist (WE) and through searching published literature. Emails were sent to the identified clinicians who were asked to independently classify the patient using free text, without stipulating a specific classification system. They were also asked to explain the reason for each classification decision.

We determined the most common classification given for each patient and calculated the % agreement (total experts giving most common classification/total experts classifying the particular photo) with 95% confidence intervals. Krippendorff’s alpha was calculated as reliability measure for agreement with a value of 1 indicating perfect agreement and 0 indicating agreement equivalent to chance. We reclassified patients based on the most given classification type. This data was used to analyze whether there was a change in the reported treatment outcomes by CL type, compared to what was reported in the original publication using logistic regression as reported previously [7]. Guidelines for Reporting Reliability and Agreement studies were followed (S1 Checklist).

## Results

A total of 26 patients were selected, with one additional patient as quality control. Among them, sixteen were originally classified as DCL, eight as MCL, one as LCL and one as *Leishmania* recidivans. These patients constituted 62% (16/26), 17% (8/47) and 5% (1/19) of the total DCL, MCL and LCL cases respectively, indicating that most classification difficulties existed for patients originally classified as DCL. From 21 invited physicians and experts, 13 (62%) responded and classified the lesions. Nine were Ethiopian dermatologists, one was an internist, one a general practitioner and two health officers with a MSc in Tropical dermatology. They came from nine different institutions. All were or had been actively involved in diagnosing and treating CL patients for at least two years, with a median of 6 years’ experience (IQR 2.5-8). Classification results are shown in Table 1. The most commonly used classifications were LCL, MCL, and DCL, although disseminated CL, *Leishmania* recidivans (either as separate classification or subtype of LCL), and mucosal leishmaniasis (ML) were also used.

**Table 1:** Classifications given by expert panel.

		Classifications				Classifications by expert panel					
ID	Fig	Original	Majority vote	Agreement n/N (%)	95% CI	LCL	MCL	DCL	Other	NA	Classification Issue identified
**Complete agreement**											
QC		LCL	LCL	13/13 (100)	77–100	13				–	None- consensus reached
**≥80% agreement**											
11		MCL	MCL	11/12 (92)	65-100	1	11			1	Lesion touching mucosa
3	2A	MCL	MCL	11/12 (92)	65-100		11		1 ML	1	MCL vs ML
13	–	DCL	MCL	11/13 (85)	58-96	1	11	1		–	Large diffuse lesions: LCL, MCL, or DCL
14		MCL	MCL	11/13 (85)	58-96	0	11		1 LCL/MCL, 1 dissCL	–	Mucosal involvement unclear
15		DCL	MCL	11/13 (85)	58-96	1	11	1		–	Multiple lesions including edge lip
16		MCL	MCL	11/13 (85)	58-96	1	11	1		–	Diffusely swollen lesion including nose/lip
5	3A	DCL	LCL	11/13 (85)	58-96	11	1	1		–	Multiple lesions on one body part
17		DCL	MCL	11/13 (85)	58-96	1	11	1		–	Lesion touching mucosa
18		MCL	MCL	10/12 (83)	55-95	1	10		1 LCL/MCL	1	Diffusely swollen lip lesion
4	2B	DCL	MCL	8/10 (80)	49-94	1	8	1		3	Large diffuse lesions: LCL, MCL, or DCL
**50-80% agreement**											
7	3D	DCL	LCL	10/13 (77)	50-92	10		2	1 dissCL	–	Multiple body parts, different sides body
6	3B/C	DCL	LCL	9/12 (75)	47-91	9		2	1 dissCL	1	Multiple body parts, same side body
19	–	DCL	MCL	9/13 (69)	42-87	2	9	2		–	Multiple lesions on different body part including edge of lip
20		DCL	DCL	9/13 (69)	42-87	1	2	9	1 MCL + DCL	–	Diffuse nodules with swollen lip involvement
21		DCL	LCL	9/13 (69)	42-87	9	2	2		–	Multiple bite LCL or DCL; Close to mucosa
24		MCL	MCL	8/12 (67)	35-90	2	8	1	1 LCL/MCL	1	Diffuse lesion over nose
22		MCL	LCL	7/11 (64)	35-84	7	4			2	Close to mucosa
9	4A	DCL	DCL	8/13 (62)	36-82	4		8	1 LCL-recidivans type	–	Lesion presentation DCL
12	5	LR	LCL	8/13 (62)	36-82	8			3 LCL-recidivans type, 2 LR	–	Recidivans vs LCL
8	3E	DCL	LCL	8/13 (62)	36-82	8	2	1	2 dissCL	–	Multiple body parts, different sides body
23		DCL	MCL	8/13 (62)	36-82	3	8	1	1 LCL/MCL	–	Diffusely swollen lesion including the nose
1	1A	MCL	LCL	7/12 (58)	32-81	7	5			1	Close to mucosa, but not actually touching
10	4B/4C	LCL	LCL	7/13 (54)	29-77	7		6		–	Lesion presentation DCL
**<50% agreement**											
25		DCL	LCL	6/13 (46)	25-75	6	2	1	1 dissCL, 3 LCL/MCL	–	Mucosal involvement unclear
2	1B	DCL	MCL	5/12 (42)	19-68	4	5	3		1	Touching mucosa (LCL vs MCL), multiple lesions on both side face (LCL vs DCL)
26		DCL	MCL	5/12 (42)	19-68	4	5	1	1 LR, 1 CL-recidivans type	1	Mucosal involvement unclear

DCL: diffuse cutaneous leishmaniasis; dissCL: disseminated cutaneous leishmaniasis; ID: patient ID, LCL: localized cutaneous leishmaniasis; LR: *Leishmania* recidivans MCL: muco-cutaneous leishmaniasis, ML: mucosal leishmaniasis, NA: not available, missing results.

Complete agreement was reached only for the patient chosen as quality control (S2 Fig), which was a clear-cut LCL case. Agreement ranged from 42-92%, with more than 80% agreement for ten patients (38%), for 13 patient (50%), 50–80% of the experts agreed, and for three patients (12%) less than half of the experts agreed. Krippendorf’s alpha was 0.069, indicating agreement was close to chance. Issues identified as main points of disagreement are discussed below.

**Fig 1:**
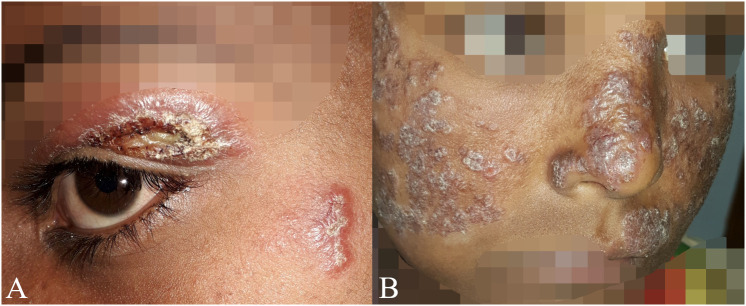
Patients with classification disagreements concerning whether the mucosa is involved or not. A) Case 1 with lesions close to the mucosa classified as localized cutaneous leishmaniasis or muco-cutaneous leishmaniasis. Seven (58%) clinicians classified it as MCL, while five (42%) classified it as LCL. B) Case 2 with lesions extending from the skin seemingly touching the mucosa but without clear mucosal involvement with swelling or infiltration. Five (42%) clinicians classified this as MCL, 4 (33%) as LCL, and 3 (25%) as DCL.

**Fig 2:**
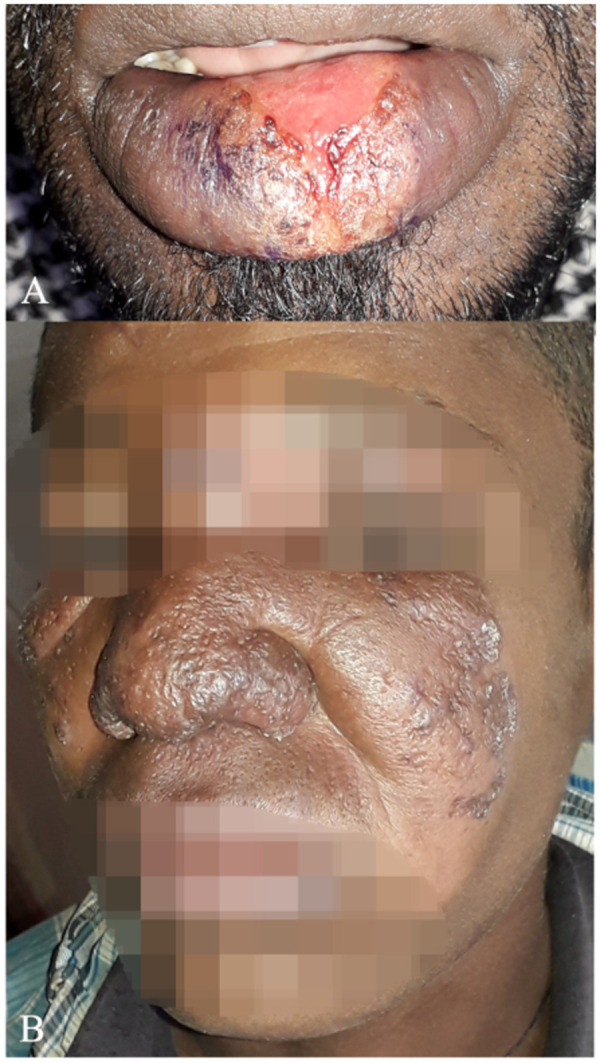
Patients with other classification disagreements concerning mucosal involvement. A) Case 3 with a lesion only affecting the lip without involving the skin. It was classified as MCL by all clinicians except one, who classified as mucosal leishmaniasis, arguing that only mucosa was involved without cutaneous lesions. B) Case 4 with large, diffusely swollen lesions classified as muco-cutaneous leishmaniasis (n = 8), diffuse cutaneous leishmaniasis (n = 1) or localized cutaneous leishmaniasis (n = 1).

There was disagreement among experts whether patients with lesions close to the mucosa (e.g., within 1 cm, without touching the mucosal border) should be classified as LCL or MCL (Fig 1A). Another point of disagreement was whether patients with lesions touching the mucosal border, but no clear mucosal involvement should be classified as LCL or MCL (Fig 1B). For example, Whether lesions confined to the mucosa without cutaneous involvement should be classified as MCL or mucosal leishmaniasis is unclear (Fig 2A).

Several patients had large diffusely swollen lesions on their face with involvement of the lip or mucosal border of the nostrils (Fig 2B). Some clinicians classified these patients as DCL, but most as MCL. One clinician highlighted that diffuse swelling of the lip without clear lesions should not be seen as mucosal involvement, since it highlights inflammation, rather than presence of parasites. This clinician therefore classified such patients as LCL, while most classified as MCL.

**Fig 3:**
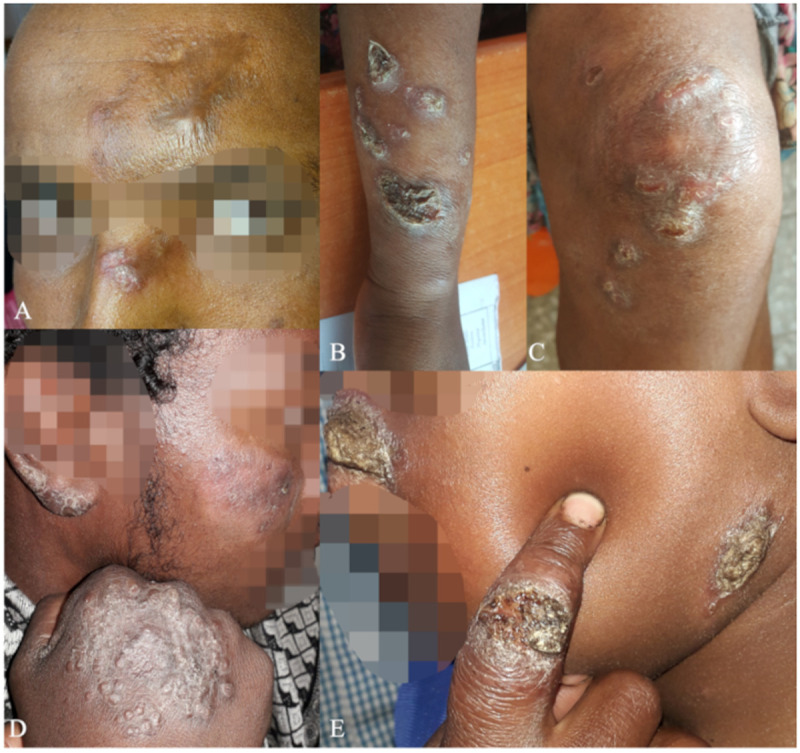
Classification disagreements for patients with multiple lesions. A) Case 5 with lesions on one body part classified as localized cutaneous leishmaniasis (n = 11), muco-cutaneous leishmaniasis (n = 1), or diffuse-cutaneous leishmaniasis (n = 1). B/C) Case 6 with lesions on the arm and leg on the same side of the body classified as localized cutaneous leishmaniasis (n = 9), diffuse cutaneous leishmaniasis (n = 2) or disseminated cutaneous leishmaniasis (n = 1). D) Case 7 with multiple lesions on different body parts classified as localized cutaneous leishmaniasis (n = 10), diffuse cutaneous leishmaniasis (n = 2) or disseminated cutaneous leishmaniasis (n = 1). E) Case 8 with multiple lesions on different body parts including the nose classified as localized cutaneous leishmaniasis (n = 8), muco-cutaneous leishmaniasis (n = 2), diffuse cutaneous leishmaniasis (n = 1) or disseminated cutaneous leishmaniasis (n = 2). It should be noted that being close to the mucosa further complicated classification.

**Fig 4:**
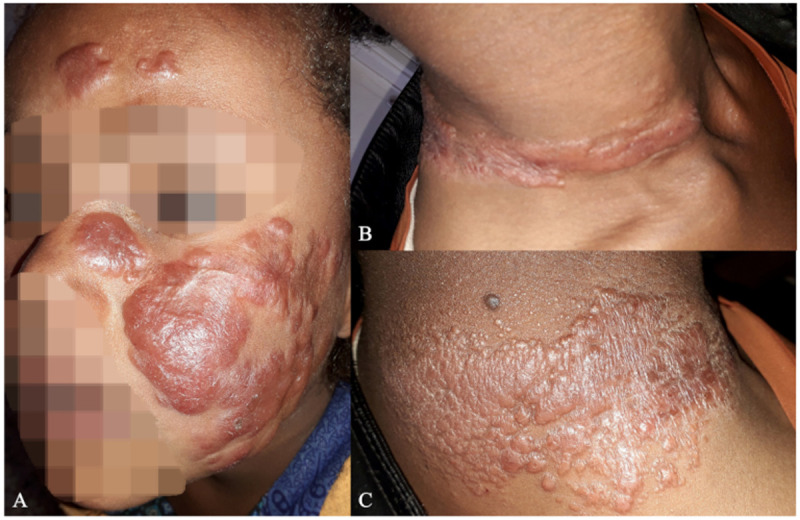
Classification disagreements for patients with certain lesion presentations. A) Case 9 with red shiny nodular plaque lesions on the cheeks classified as diffuse cutaneous leishmaniasis (n = 8), localized cutaneous leishmaniasis (n = 4), (of which one specified recidivans type).). B/C) Case 10 with erythematous nodular plaques on the neck classified as localized cutaneous leishmaniasis (n = 7) or diffuse cutaneous leishmaniasis (n = 6).

It was unclear whether patients with multiple lesions on multiple body parts should be classified as LCL or DCL. Patients with multiple lesions present with different lesion distributions: 1) one body part (Fig 3A), 2) limited to the face but on both sides (Fig 1B), 3) one side of two body parts (e.g., left arm (Fig 3B) and left leg (Fig 3C)) and 4) on multiple body parts on both sides (Fig 3D and 3E). The first three groups were mostly classified as LCL. However, for patients with lesions on multiple sides and multiple body parts (Fig 3D and 3E), they were increasingly classified as DCL or disseminated CL.

Whether certain lesion features such as diffuse nodular lesions without surface changes (Fig 4) should lead to classification of DCL was a point of contention for two cases.

**Fig 5:**
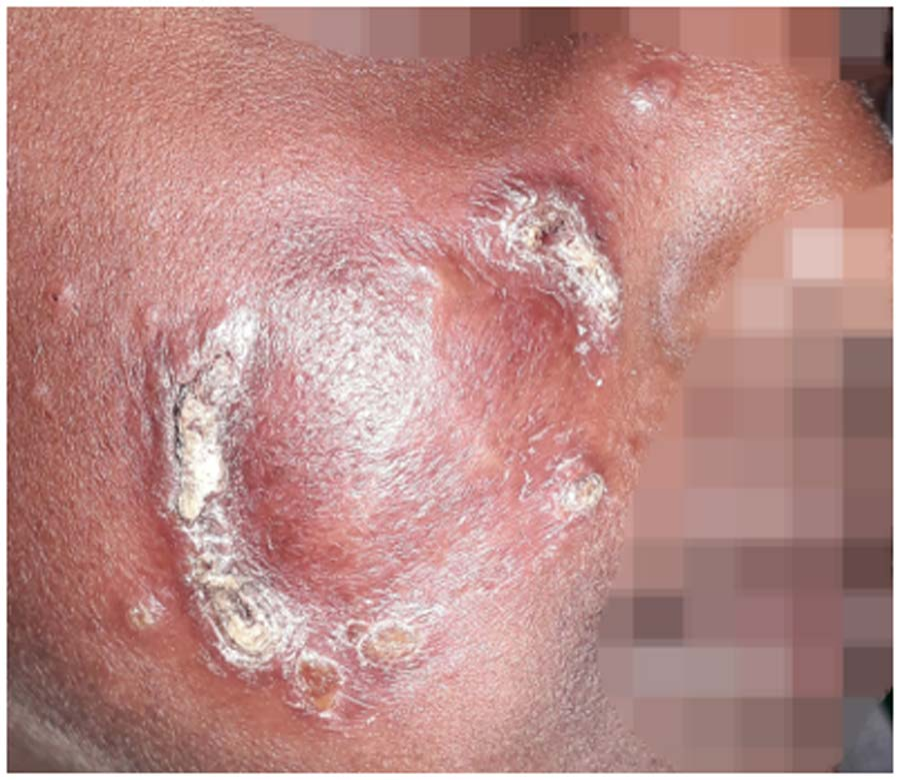
Classification disagreements for a patient with an active lesion around a healed scar. Case 11 with active lesions around a centrally healed scar with additional satellite lesions classified as localized cutaneous leishmaniasis (n = 8) as Leishmania recidivans (n = 2) or as LCL – recidivans subtype (n = 3).

Identifying *Leishmania* recidivans is not uniformly understood as highlighted by case 11 who had a lesion with an active border around a centrally healed scar (Fig 5), but was still classified as LCL by the majority of experts.

For 16 of the 26 (62%) selected patients, our experts had a different majority vote leading to reclassification compared to the original study [7] classification (Table 1). This was mostly true for patients originally classified as DCL. Seven patients were reclassified from DCL to MCL, and six from DCL to LCL. According to the expert panel majority vote, the number of DCL patients in the original manuscript decreased by 50% to a total of 13. Outcomes by classification in the original classification and after reclassification are shown for both study sites combined, and for Boru Meda and Gondar separately (S1–S3 Tables, respectively). Originally, outcomes were significantly different for the three classifications in Boru Meda, with the highest cure rate (91%) found for DCL patients, and only 29% cure for LCL patients. After updating the classifications according to the panel majority vote, outcomes remained significantly different but cure rate for DCL was now 75%, MCL 82%, and LCL 50% (S2 Table).

## Discussion

This study highlights significant disagreement in classification of CL among clinical experts within a single country. The lack of clear, standardized definitions leads to varying interpretations and classifications, which can introduce bias in published research. Sixteen of the 26 selected cases from our original publication would be classified differently according to the majority classification of experts. Importantly, there was no agreement on the classification for any of these 26 cases. Consistent classification is also important from a public health perspective, as the reported proportions of MCL and DCL cases influence decisions on drug purchasing and planning of inpatient treatment facilities.

Classification of LCL seems most straightforward, as only one patient originally classified as LCL was selected as ‘difficult patient.’ This was confirmed by the fact that there was complete agreement for the LCL patient included as a quality control. Classification becomes challenging for patients who have lesions close to the mucosa which some call MCL or when there are multiple lesions or lesions with certain features, leading some clinicians to consider DCL. The existing literature in Ethiopia states that LCL develops on the site of the sandfly bite [8,10]*,* that LCL can be large [10] and can consist of multiple lesions [6,8,10]*,* and therefore there seems agreement that LCL is a lesion without mucosal involvement that can be single or multiple.

Our expert panel showed that the term MCL is widely used but interpreted differently, with certain clinicians classifying lesions on the outer vermillion part of the lips, close to the eye, and close or on the alar rim as MCL, although these regions are anatomically not true mucosa. Expert classification also highlighted there is no consensus whether lesions close to the mucosa, just touching the mucosa or only superficially involving the lip or muco-cutaneous border should be classified as MCL. A recent pre-print by Mohammed *et al*. about interobserver agreement of assessing CL lesions in a single hospital in Ethiopia confirms this issue, as for six out of the 12 assessed cases, clinicians could not agree on whether patents were LCL or MCL [11]. Although several reports mentioned that true ‘mucosal’ lesions without concomitant cutaneous involvement are rare in Ethiopia, one of our cases was such an example and was classified as ML by one clinician. Mohammed *et al* [11] also describe two cases where clinicians disagreed on whether are MCL or ML, which further raises the question whether patients with lesions confined to the mucosa should be classified separately as ML or included under MCL.

In the Ethiopian literature, the term MCL is also widely employed, often using terminology more fitting NW MCL. As a result, Ethiopian MCL is commonly described as having marked involvement of the naso-oral and pharyngeal mucosa, with destructive and mutilating effects [9,10,12,13]. However, early studies clearly noticed that although nasal lesions are common in Ethiopia, infiltration of parasites does not usually extend beyond the muco-cutaneous junction, and does not cause destructive lesions involving cartilage, the pharynx, and palate [14–18]. In NW MCL, lesions typically develop after hematogenous or lymphatic spread of primary cutaneous lesions sometimes years later. The pathophysiology of MCL in Ethiopia seems to be radically different. Most lesions considered as MCL are primary lesions having concomitant mucosal and cutaneous involvement, with lesions either extending from the mucosa after sandfly bites directly on the mucosal borders [6,8] to the skin [13,17] or vice versa [6,13] rather than resulting from metastatic spread. Interestingly, the WHO’s report on Leishmaniasis control [19] indicates that the term MCL should only be applied to NW CL.

Most confusion in classifying CL in Ethiopia relates to DCL. In practice, there was lack of consensus on whether large but single diffusely swollen lesions on the face with or without mucosal involvement should be classified as DCL, whether patients with multiple lesions are automatically considered to be DCL spread through the blood or when to consider them to be due to multiple bites. Additionally, whether certain lesion characteristics (e.g., lesions resembling lepromatous leprosy) classify a patient as having DCL was unclear, and how to distinguish between disseminated and DCL patients clinically.

DCL was first described in Ethiopia in detail by Bryceson in 1969 [20]. He described typical characteristics of DCL which relate to clinical findings as well as pathophysiology - these characteristics are also still referred to by most more recent literature and include the following: 1) an initial local lesion often on a limb that eventually disseminates [21,22] to other body parts and can involve large areas [6,8,10,21,23,24], 2) nodular lesions which do not ulcerate [8,10,21,23–29] and self-heal [8,18,21,30], 3) a superabundance of parasite [10,21,26,27,29,31], 4) slow disease progression which becomes chronic [8,10,21,27,29], 5) poor response to antimony treatment [8,22,26], 6) resembling lepromatous leprosy [8,10,18,26,27]. However, he also described that not every patient classified as DCL needs to fit all those criteria.

*Leishmania* recidivans was used as classification by only a few clinicians, despite inclusion of a typical case with active lesions at the edge of a healed *Leishmania* scar (Fig 5A). This case was mostly classified as LCL indicating that more clarity is needed. Not much has been published about this form and it is also not mentioned in the national guidelines, although a few reports of a limited number of cases exist [12,13,32].

We did not aim to provide a representative overview to which extent consensus for classification of CL exists. Rather, we wanted to document how experts classified a selection of CL cases that were purposefully selected as difficult to classify, to outline points of disagreement. We also acknowledge that our panel of experts may not be representative, as only experts interested to participate were included. Other limitations are the small number of patients included, and inability to look at other potential contributing factors such as species and geographical differences. Due to the small sample size, we were unable to look at factors which may have affected classification, such as level of experience in treating CL, the level of training and regional trends which could arise from people being trained by the same individuals or institutes. Lastly, classifying using photographs may not fully reflect classification as it occurs in practice while examining patients, especially when deciding whether the mucosa is involved.

We used the classification given by the majority to reclassify the cases from our original manuscript to see to which extent disagreements could affect reported findings. The proportion of DCL patients reduced drastically after reclassification, and afterwards, DCL no longer had the highest proportion of cure. This indicates that consistent classification is needed across the country as we move towards larger clinical trials.

Clear reproducible guidelines should be developed if the terms LCL, MCL, DCL and *Leishmania* recidivans will continue to be used in Ethiopia, to allow for consistent classification. An attempt has been made within the SHARP project [33], which proposes a new classification system that separates MCL from mucosal leishmaniasis, and subdivides LCL into localized and multi-regional LCL based on clear criteria. However, it remains unclear whether this new system leads to more consensus and is in line with how the majority of clinicians classify patients. Consensus meetings with dermatologist and CL experts should be undertaken to move this initiative forward, and ideally lead to clear classification algorithms which can be used by non-experts.

Alternatively, it could be argued that the common classifications currently used globally may not always be applicable to every setting – including Ethiopia. This seems especially the case for MCL, which appears to be radically different from the hyperinflammatory, purely mucosal metastatic MCL that is seen in the NW. Parasitological and immunological research may shed more light on whether patients with mucosal involvement are inherently different from those with cutaneous lesions alone. A recent study done in Ethiopia found no differences in parasite genetics, and immunological signature (measured by cytokines and chemokines) between clinical classifications. However, authors used a new classification system with contained CL and spreading CL as categories, which does not match what clinicians in other hospitals use, and specific definitions for classification are not clearly described [34].

Ideally, classification is linked to clinical decision-making. Currently, patients classified as MCL or DCL are typically started on systemic treatment, as they are assumed to have poorer outcomes than LCL patients. One of the main reasons to treat MCL systemically, is that it is assumed to not heal spontaneously, and potentially cause extensive and disfiguring lesions, as mentioned by the Ethiopian leishmaniasis guidelines.[8] However, this seems to have been extrapolated from the Latin American setting, and Ethiopian evidence is emerging that some lesions classified as MCL were successfully treated with local therapy [35], and we confirmed here again that treatment outcomes are not necessarily worse in those with MCL or DCL also after reclassification [7]. Currently, most clinicians group very small lesions on the lip together with extensive lesions with mucosal destruction. Although both have mucosal involvement, each probably needs a different treatment approach and has a different prognosis.

Classification assumes similarity within the group, - not always evident in the Ethiopian setting and should be linked to decision making. Therefore, an alternative would be to move away from unclear and heterogenous classification criteria of LCL, MCL and DCL, but rather to move towards a more clinically oriented classification that simply describes whether local or systemic treatment is needed. A similar approach is already recommended in the European context and American Infectious Diseases Society (IDSA) guidelines, which classify patients as simple when they can be treated with local treatment or complex when systemic treatment is required [36].

## Supporting information

S1 Fig Typical examples of localized cutaneous leishmaniasis, muco-cutaneous leishmaniasis and diffuse cutaneous leishmaniasis. (DOCX)

S2 Fig Case 0 with consensus classification as localized cutaneous leishmaniasis. (DOCX)

S1 Checklist GRRAS checklist for reporting of studies of reliability and agreement. (DOCX)

S1 Table Day 90 treatment outcomes by classification overall according before and after reclassification. (DOCX)

S2 Table Day 90 treatment outcomes by classification for Boru Meda before and after reclassification. (DOCX)

S3 Table Day 90 treatment outcomes by classification for Gondar before and after reclassification. (DOCX)

S1 File Raw data. (XLSX)
