# Supplementary material for: Ambiguities in cutaneous leishmaniasis classification and the need for consensus: Experience from Ethiopia
Source: PLoS Negl Trop Dis. 2025 Aug 22;19(8):e0013458. doi: 10.1371/journal.pntd.0013458 (PMC12396759; doi:10.1371/journal.pntd.0013458)
Supplement: S1 Fig — (DOCX) [file pntd.0013458.s001.docx]

**S1 Fig.** Typical presentations of localized cutaneous leishmaniasis, muco-cutaneous leishmaniasis, and diffuse cutaneous leishmaniasis

**
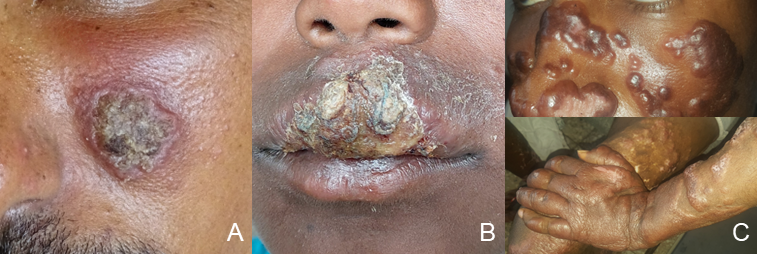
**A. Localized cutaneous leishmaniasis (LCL): Clearly demarcated nodule or plaque with possible crustation, erythema and ulceration on the skin without involving the mucosa. Appears on the site of the sand-fly bite, can be single or multiple. B. Muco-cutaneous leishmaniasis (MCL): Primary lesion on the mucosa or contagious spread from adjacent skin producing swelling of lips or nasal mucosa. Mostly remains confined to the borders of the nose and mouth. C. Diffuse cutaneous leishmaniasis (DCL): Multiple papular, nodular or plaque, non-ulcerated lesions involving multiple body parts, presumably from spread through lymphohematogenous spread
