# Supplementary figures and images for: Ambiguities in cutaneous leishmaniasis classification and the need for consensus: Experience from Ethiopia
Source: PLoS Negl Trop Dis. 2025 Aug 22;19(8):e0013458. doi: 10.1371/journal.pntd.0013458 (PMC12396759; doi:10.1371/journal.pntd.0013458)

**S2 Fig.** Case 0 with consensus classification as localized cutaneous leishmaniasis


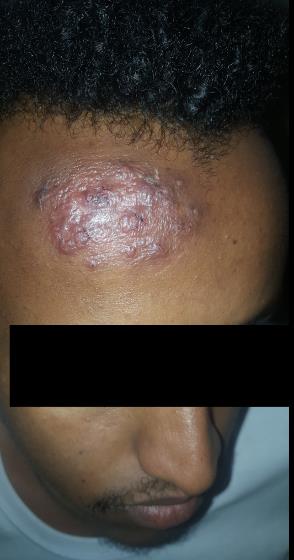

Supplement: S2 Fig — (DOCX) [file pntd.0013458.s002.docx]
