# Supplementary material for: Ambiguities in cutaneous leishmaniasis classification and the need for consensus: Experience from Ethiopia
Source: PLoS Negl Trop Dis. 2025 Aug 22;19(8):e0013458. doi: 10.1371/journal.pntd.0013458 (PMC12396759; doi:10.1371/journal.pntd.0013458)
Supplement: S1 Checklist — (DOCX) [file pntd.0013458.s003.docx]

**S1 GRRAS checklist for reporting of studies of reliability and agreement**

| **Section** | **Item #** | **Checklist item** | **Reported on page #** |
| --- | --- | --- | --- |
| Title/Abstract | 1 | Identify in title or abstract that interrater/intrarater  reliability or agreement was investigated. | Abstract: objective |
| Introduction | 2 | Name and describe the diagnostic or measurement device of interest explicitly. | Agreement in classification given, specified in introduction, last paragraph |
|  | 3 | Specify the subject population of interest. | ‘Difficult CL cases’, specified in introduction, last paragraph |
|  | 4 | Specify the rater population of interest (if applicable). | Ethiopian CL experts, specified in introduction, last paragraph |
|  | 5 | Describe what is already known about reliability and  agreement and provide a rationale for the study (if applicable). | Last paragraph introduction |
| Methods | 6 | Explain how the sample size was chosen. State the determined number of raters, subjects/objects, and replicate observations. | Methods, 5th paragraph |
|  | 7 | Describe the sampling method. | Methods, 5th paragraph |
|  | 8 | Describe the measurement/rating process (e.g. time interval between repeated measurements, availability  of clinical information, blinding). | Methods, 5th paragraph |
|  | 9 | State whether measurements/ratings were conducted independently. | Yes, see methods, 5th paragraph |
|  | 10 | Describe the statistical analysis. | Last paragraph methods |
| Results | 11 | State the actual number of raters and subjects/objects  which were included and the number of replicate observations which were conducted. | First paragraph results. |
|  | 12 | Describe the sample characteristics of raters and  subjects (e.g. training, experience). | First paragraph results. |
|  | 13 | Report estimates of reliability and agreement including measures of statistical uncertainty. | Table 1 |
| Discussion | 14 | Discuss the practical relevance of results. | Throughout discussion |
| Auxiliary material | 15 | Provide detailed results if possible (e.g. online). | See Table S1,2,3. Supporting figure S1 and S2. |
| *Version based on Table I in: Kottner J, Audigé L, Brorson S, Donner A, Gajeweski BJ, Hróbjartsson A, Robersts C, Shoukri M, Streiner DL. Guidelines for reporting reliability and agreement studies (GRRAS) were proposed. J Clin Epidemiol. 2011;64(1):96-106* | | | |
