# Supplementary material for: Ambiguities in cutaneous leishmaniasis classification and the need for consensus: Experience from Ethiopia
Source: PLoS Negl Trop Dis. 2025 Aug 22;19(8):e0013458. doi: 10.1371/journal.pntd.0013458 (PMC12396759; doi:10.1371/journal.pntd.0013458)
Supplement: S1 Table — (DOCX) [file pntd.0013458.s004.docx]

**S1 Table.** Day 90 treatment outcomes by classification overall according before and after reclassification

| **Original classification** | **LCL^a^**  **N=12** | | **MCL**  **N=29** | | **DCL**  **N=17** | |  |
| --- | --- | --- | --- | --- | --- | --- | --- |
|  | **n (%)** | **95% CI** | **n (%)** | **95% CI** | **n (%)** | **95% CI** | **P** |
| Cure | 4 (18.7) | 3.0 – 49.6 | 19 (41.7) | 12.7 – 76.4 | 10 (45.1) | 15.9 – 73.4 | 0.100 |
| Good improvement | 3 (32.8) | 2.6 – 83.7 | 9 (48.7) | 12.3 – 84.1 | 4 (31.4) | 4.7 – 72.2 |  |
| Partial Improvement | 0 (0) | - | 0 (0) | - | 0 (0) | - |  |
| No improvement | 0 (0) | - | 0 (0) | - | 0 (0) | - |  |
| Relapse | 5 (48.4) | 6.0 – 91.9 | 1 (9.7) | 0.4 – 41.6 | 3 (23.6) | 2.8 – 66.0 |  |
| **Reclassification** | **LCL^a^**  **N=19** | | **MCL^b^**  **N=32** | | **DCL**  **N=8** | |  |
|  | **n (%)** | **95% CI** | **n (%)** | **95% CI** | **n (%)** | **95% CI** |  |
| Cure | 8 (28.1) | 0 – 65.8 | 22 (42.3) | 0 – 72.1 | 3 (30.2) | 0 – 68.3 | 0.200 |
| Good improvement | 6 (40.8) | 0.9 – 75.9 | 9 (52.5) | 23.4 – 99.8 | 2 (29.8) | 0 – 69.9 |  |
| Partial Improvement | 0 (0) | - | 0 (0) | - | 0 (0) | - |  |
| No improvement | 0 (0) | - | 0 (0) | - | 0 (0) | - |  |
| Relapse | 5 (31.1) | 0 – 64.2 | 1 (5.3) | 0 – 19.2 | 3 (40.0) | 0 – 98.4 |  |
| ^a^All proportions are adjusted by site. ^b^One case had equal classifications as LCL and MCL and was grouped as MCL here. CI: confidence interval; DCL: diffuse cutaneous leishmaniasis; LCL: localized cutaneous leishmaniasis; MCL: muco-cutaneous leishmaniasis | | | | | | | |
