# Supplementary material for: Ambiguities in cutaneous leishmaniasis classification and the need for consensus: Experience from Ethiopia
Source: PLoS Negl Trop Dis. 2025 Aug 22;19(8):e0013458. doi: 10.1371/journal.pntd.0013458 (PMC12396759; doi:10.1371/journal.pntd.0013458)
Supplement: S2 Table — (DOCX) [file pntd.0013458.s005.docx]

**S2 Table.** Day 90 treatment outcomes by classification for Boru Meda before and after reclassification

| **Original classification** | **LCL**  **N=7** | | **MCL^a^**  **N=24** | | **DCL**  **N=11** | |  |
| --- | --- | --- | --- | --- | --- | --- | --- |
|  | **n (%)** | **95% CI** | **n (%)** | **95% CI** | **n (%)** | **95% CI** | **P** |
| Cure | 2 (28.6) | 0–68.4 | 19 (79.2) | 66.7–95.7 | 10 (90.9) | 81.8–100 | 0.005 |
| Good improvement | 3 (42.9) | 14.3–82.6 | 5 (20.8) | 8.3–37.4 | 0 (0) | 0–15.1 |  |
| Partial Improvement | 0 (0) | 0–39.8 | 0 (0) | 0–16.5 | 0 (0) | 0–15.1 |  |
| No improvement | 0 (0) | 0–39.8 | 0 (0) | 0–16.5 | 0 (0) | 0–15.1 |  |
| Relapse | 2 (28.6) | 0–68.4 | 0 (0) | 0–16.6 | 1 (9.1) | 0–24.2 |  |
| **Reclassification** | **LCL**  **N=12** | | **MCL^a^**  **N=27** | | **DCL**  **N=4** | |  |
|  | **n (%)** | **95% CI** | **n (%)** | **95% CI** | **n (%)** | **95% CI** | **P** |
| Cure | 6 (50.0) | 25.0 – 78.3 | 22 (81.5) | 70.4 – 96.5 | 3 (75.0) | 50.0 – 100 | 0.045 |
| Good improvement | 4 (33.3) | 8.3 – 61.6 | 5 (18.5) | 7.4 – 33.5 | 0 (0) | 0 – 38.4 |  |
| Partial Improvement | 0 (0) | 0 – 28.3 | 0 (0) | 0 – 15.0 | 0 (0) | 0 – 38.4 |  |
| No improvement | 0 (0) | 0 – 28.3 | 0 (0) | 0 – 15.0 | 0 (0) | 0 – 38.4 |  |
| Relapse | 2 (16.7) | 0 – 45.0 | 0 (0) | 0 – 15.0 | 1 (25.0) | 0 – 63.4 |  |
| ^a^One case had equal classifications as LCL and MCL and was grouped as MCL here. CI: confidence interval; DCL: diffuse cutaneous leishmaniasis; LCL: localized cutaneous leishmaniasis; MCL: muco-cutaneous leishmaniasis | | | | | | | |
