# Supplementary material for: Ambiguities in cutaneous leishmaniasis classification and the need for consensus: Experience from Ethiopia
Source: PLoS Negl Trop Dis. 2025 Aug 22;19(8):e0013458. doi: 10.1371/journal.pntd.0013458 (PMC12396759; doi:10.1371/journal.pntd.0013458)
Supplement: S3 Table — (DOCX) [file pntd.0013458.s006.docx]

**S3 Table.** Day 90 treatment outcomes by classification for Gondar before and after reclassification

| **Original classification** | **LCL**  **N=5** | | **MCL**  **N=5** | | **DCL**  **N=6** | |  |
| --- | --- | --- | --- | --- | --- | --- | --- |
|  | **n (%)** | **95% CI** | **n (%)** | **95% CI** | **n (%)** | **95% CI** | **P** |
| Cure | 2 (40) | 20.0–91.2 | 0 (0) | 0–30.5 | 0 (0) | 0–44.8 | 0.054 |
| Good improvement | 0 | 0–51.1 | 4 (80.0) | 60.0–100 | 4 (66.7) | 50.0–100 |  |
| Partial Improvement | 0 | 0–51.1 | 0 (0) | 0–30.5 | 0 (0) | 0–44.8 |  |
| No improvement | 0 | 0–51.1 | 0 (0) | 0–30.5 | 0 (0) | 0–44.8 |  |
| Relapse | 3 (60) | 0.4–100 | 1 (20.0) | 0–50.5 | 2 (33.3) | 167–78.2 |  |
| **Reclassification** | **LCL**  **N=7** | | **MCL^a^**  **N=5** | | **DCL**  **N=4** | |  |
|  | **n (%)** | **95% CI** | **n (%)** | **95% CI** | **n (%)** | **95% CI** | **P** |
| Cure | 2 (28.6) | 0 – 69.7 | 0 (0) | 0 – 32.6 | 0 (0) | 0 – 58.4 | 0.460 |
| Good improvement | 2 (28.6) | 0 – 69.7 | 4 (80.0) | 60.0 – 100 | 2 (50.0) | 25.0 – 100 |  |
| Partial Improvement | 0 (0) | 0 – 41.2 | 0 (0) | 0 – 32.6 | 0 (0) | 0 – 58.4 |  |
| No improvement |  |  | 0 (0) | 0 – 32.6 | 0 (0) | 0 – 58.4 |  |
| Relapse | 3 (42.9) | 14.3 – 84.0 | 1 (20.0) | 0 – 52.6 | 2 (50.0) | 25.0 - 100 |  |
| CI: confidence interval; DCL: diffuse cutaneous leishmaniasis; LCL: localized cutaneous leishmaniasis; MCL: muco-cutaneous leishmaniasis | | | | | | | |
